# Application of Polyhydroxyalkanoates in Medicine and the Biological Activity of Natural Poly(3-Hydroxybutyrate)

**DOI:** 10.32607/20758251-2019-11-2-4-16

**Published:** 2019

**Authors:** A. P. Bonartsev, G. A. Bonartseva, I. V. Reshetov, M. P. Kirpichnikov, K. V. Shaitan

**Affiliations:** Faculty of Biology, M.V. Lomonosov Moscow State University, Leninskie Gory 1, bldg. 12, Moscow, 119234, Russia; A.N. Bach Institute of Biochemistry, Research Center of Biotechnology of the Russian Academy of Sciences, Leninsky Ave. 33, bldg. 2, Moscow, 119071, Russia; Sechenov First Moscow State University, Trubetskaya Str. 8, bldg. 2, Moscow, 119991, Russia

**Keywords:** polyhydroxyalkanoates, poly(3-hydroxybutyrate), biosynthesis, biomimetics, biodegradation, biocompatibility, regenerative medicine

## Abstract

Biodegradable and biocompatible polymers, polyhydroxyalkanoates (PHAs), are actively used in medicine to produce a wide range of medical devices and dosage formulations. The medical industry mainly utilizes PHAs obtained by chemical synthesis, but interest in the medical application of natural PHAs obtained biotechnologically is also growing. Synthetic PHAs are the biomimetic analogs of bacterial poly(3-hydroxybutyrate) (PHB) and other natural PHAs. This paper addresses the issue of the presence of biological activity in synthetic and natural PHAs (stimulation of cell proliferation and differentiation, tissue regeneration) and their possible association with various biological functions of PHB in bacteria and eukaryotes, including humans.

## INTRODUCTION

**Fig. 1:**
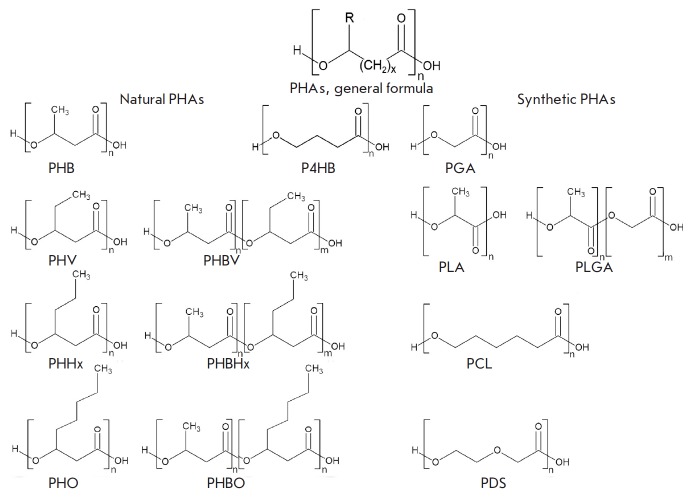
The general formula for polyhydroxyalkanoates and the structural formulas for a series of natural and synthetic polyhydroxyalkanoates for biomedical applications. Abbreviations: PHB – poly(3-hydroxybutyrate); PHV – poly(3-hydroxyvalerate); PHBV – poly(3-hydroxybutyrate-co-3-hydroxyvalerate); PHHx – poly(3-hydroxyhexanoate); PHBHx – poly(3-hydroxybutyrate-co-3-hydroxyhexanoate); PHO – poly(3-hydroxyoctanoate); PHBO – poly(3-hydroxybutyrate-co-3-hydroxyoctanoate); P4HB – poly(4-hydroxybutyric) acid (poly(4-hydroxybutyrate)); PGA – poly(2-hydroxyacetic) acid (polyclycolic acid, polyglycolide); PLA – poly(2-hydroxypropanoic) acid (polylactic acid, polylactide); PLGA – poly(lactic-co-glycolic) acid (polylactide-co-glycolide); PCL – poly(6-hydroxycaprolactone); PDS – poly(*p*-dioxanone)

Polyhydroxyalkanoates (PHAs) are biodegradable polyesters of hydroxycarbonic acids which are produced by either chemical synthesis or bacterial biosynthesis. Since the early 21^st^ century, there has been growing interest in studying these polymers and introducing them in medical practice. Synthetic poly(2-hydroxypropanoic) (polylactic (PLA), polylactides) acid and poly(2-hydroxyacetic) (polyglycolic) acid ((PGA), polyglycolides), poly(6-hydroxycaprolactone) (PCL) and natural poly(3-hydroxybutyric) acid (PHB, poly(3-hydroxybutyrate)); poly(4-hydroxybutyric) acid (P4HB), poly(3-hydroxyvaleric) acid (PHV, poly(3-hydroxyvalerate)), poly(3-hydroxyhexanoate) (PHHx), and their copolymers and polymers with a similar structure, such as poly(*p*-dioxanone) (PDS) (*Fig. 1*), are currently used both in research and clinical practice. These polymers have a similar chemical structure and, therefore, similar physicochemical and biomedical properties: they can be biodegraded in the organism without toxic product formation, are biocompatible with human organs and tissues, exhibit optimal physic ochemical properties (thermoplasticity, relatively high hydrophobicity, specific diffusion properties, relatively high strength, and flexibility). Furthermore, they can be produced through efficient technological processes. Such a unique combination of properties by these polymers contributes to their wide use and introduction in medical practice [1–4].

## APPLICATION OF POLYHYDROXYALKANOATES IN MEDICINE

**Fig. 2:**
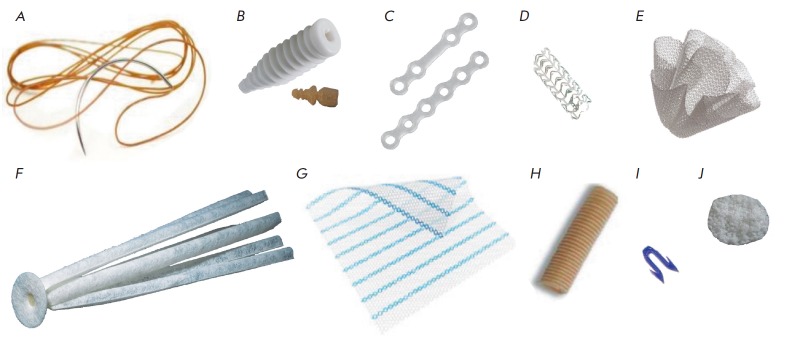
Medical devices based on synthetic and natural PHAs used in medical practice or currently being developed. *A *– PGA-based bioresorbable sutures (Ethicon, Johnson & Johnson, USA); *B *– OsteotwinTM bioresorbable interference screw for bone fixation based on PLA with a plasticizing agent (Biomatlante, France); *C *– LactoSorb® PLGA-based bioresorbable plates for bone fixation (Biomet, USA); *D *– ABSORB PLA-based bioresorbable coronary stent (Abbott, USA); *E *– Phasix Plug P4HB-based bioresorbable woven plug endoprosthesis for hernioplasty (C.R. Bard Inc., USA); *F *– Gore Bio-A fistula plug PLA-based bioresorbable plug endoprosthesis for coloproctological applications PLA (W. L. Gore & Associates Inc., USA); *G *– Ultrapro Advanced™ partially resorbable mesh endoprosthesis for hernioplasty based on a woven material made of polypropylene monofilaments and PLGA (Ethicon, Johnson & Johnson, USA); *H *– GEM Neurotube mesh tube based on woven PGA material for nerve fusion (Synovis Micro Companies Alliance, USA); *I *– PLA-based bioresorbable staple for an automated skin and soft tissue stapling device (Ethicon, Johnson & Johnson, USA); *J *– ElastoPHB PHBV-based bioresorbable biopolymeric membrane for repairing soft and cartilage tissue defects (BIOMIR Service JSC, Krasnoznamensk, Russia)

PHAs began being widely utilized in medicine as early as in the 1970s. Thus, the first biodegradable Vicryl surgical suture material produced from chemically synthesized polymers appeared on the market of medicinal products back in 1974. Various products made of PHAs are either currently in use or being developed (biodegradable surgical staples, screws, plates, pins and cords, bioresorbable suture material and skin staples, wound and burn dressings, membranes for periodontal guided regeneration, surgical mesh endoprostheses, patches for surgical repair of intestinal and pericardial defects, mesh plugs for coloproctological applications and hernioplasty, vascular prosthetic implants, coronary stents, mesh tubes for nerve regeneration, artificial heart valves, and other medical devices). PHAs are also used in pharmaceutics as components of novel dosage forms and impart such properties as targeted delivery, prolonged activity, reduced toxicity, and enhanced stability to them [1–4] (*Fig. 2*).

All members of the PHA family are characterized by a unique combination of properties. However, synthetic PHAs (sPHAs) such as poly(2-hydroxypropanoic) acid (polylactic acid or polylactide), poly(2-hydroxyacetic) acid (polyglycolic acid or polyglycolide) and their copolymers–poly(lactic-co-glycolic) acids (polylactide-co-glycolides) (PLGAs), poly(6-hydroxycaprolactone), and poly(*p*-dioxanone) – are those most typically used in medicine (*Fig. 1*). The reason behind this is the larger scale application of chemical synthesis in the production of medical polymers and earlier development of a method for the industrial-scale production of sPHAs (PLA, PGA, PCL and their copolymers), earlier certification, conduct of preclinical and clinical trials, and introduction of these polymers in clinical practice (in the 1970s–80s). An important role was also played by the fact that these polymers are very convenient to use (in particular, due to their rapid biodegradation in human tissues) [4-6].

However, PLA, PGA, and their copolymers are synthetic analogs of natural polyhydroxyalkanoates, poly(3-hydroxyalkanoates) (nPHAs). Although synthetic PHAs (including PLA, PGA, PLGA, and PCL) are quite often referred to as biopolymers, as implied by their biodegradability and biocompatibility, it is not fully accurate to use this term, since what are usually referred to as biopolymers are polymeric metabolic by-products of living organisms (bacteria, plants, fungi, and animals); i.e., natural biomacromolecules [7]. Hence, poly(3-hydroxyalkanoates) are reserve polymers in many bacterial species [1], while sPHAs (PLA, PGA, PLGA, PCL, etc.) are not found in nature [4, 8]. Although copolymers of poly(3-hydroxybutyrate) and polylactic acid have been synthesized using genetic engineering techniques employing bacterial strains [9, 10], this only provides additional evidence of their artificial origin. Nevertheless, these polymers also share key properties, although with important distinctions that have been outlined above.

Natural poly(3-hydroxyalkanoates) are polyesters of 3-hydroxyalkanoic acids; therefore, PHB is a linear polyester of (R)-3-hydroxybutyric acid (*Fig. 1*). The distinctions between different nPHAs are a result of the presence of a side radical: poly(3-hydroxybutyrate), poly(3-hydroxyvalerate), poly(3-hydroxyhexanoate), poly(3-hydroxyoctanoate), etc. (*Fig. 1*). All these compounds differ rather significantly in their physicochemical properties, such as crystallinity, the melting point and glass transition temperature, hydrophobicity, plasticity, the Young’s modulus, etc. It is important to mention that bacterial biosynthesis typically results in not pure homopolymers of poly(3-hydroxyvalerate), poly(3-hydroxyhexanoate), and other, longer chain PHA monomers but rather in their block copolymers with PHB: poly(3-hydroxybutyrate-co-3-hydroxyvalerate) (PHBV), poly(3-hydroxybutyrate-co-3-hydroxyhexanoate) (PHBHx), poly(3-hydroxybutyrate-co-3-hydroxyvalerate-co-3-hydroxyhexanoate) (PHBVHx), (poly(3-hydroxybutyrate-co-3-hydroxyoctanoate (PHBO), poly(3-hydroxybutyrate-co-4-hydroxybutyrate) (PHB4HB), etc. However, the properties of these copolymers differ significantly from those of PHB and substantially depend on the monomeric composition of the copolymer [11–13].

A technical approach is usually employed to study the biomedical properties (including biological activity) of various PHAs: one of the materials intended for the development of a certain medical device is tested. But what if we analyze the biomedical properties of PHA using PHB as a natural progenitor of almost all the PHAs utilized in medicine in terms of all the functions that this polymer possesses when occurring in nature? In other words, we are going to use biomimetics, an interesting biological discipline [14]. There is all the more reason for this as the biomimetic approach has recently been increasingly in use in the study of various polymers [15].

## THE BIOLOGICAL ACTIVITY OF POLYHYDROXYALKANOATES

**Biocompatibility of polyhydroxyalkanoates and their biological activity**

Natural biopolymers, such as proteins and peptides, polysaccharides, lipids, nucleic acids, polyprenols, and their copolymers, typically exhibit intense biological activity that is directly related to their specialized functions: enzymatic, regulatory, signaling, defense, transport, etc. Furthermore, even biopolymers such as lipopolysaccharides or pectins playing “neutral” functions (structure-forming or reserve ones) can also exhibit a pronounced biological activity [16]. Therefore, medical products or pharmaceuticals based on some of these biopolymers (collagen, chitosan, and polylysine) may have a biological activity that is sometimes undesirable (e.g., immunotoxicity) [17]. However, despite the intensive research that is currently underway, the question regarding the biological activity of both synthetic and natural PHAs remains rather controversial and insufficiently studied. On the other hand, the wide application of PHAs in medicine is largely a result of the fact that they are highly biocompatible and are either non- or low-toxic, which does not preclude the biological activity in these polymers [17]. Meanwhile, biodegradability is the key reason why PHAs are utilized in medicine. However, the process of polymer biodegradation implies that there is intensive interaction between the polymer and the surrounding living cells and tissues (that often are involved in this process) and that the cells and tissues are affected not only by the polymer, but also by its biodegradation products (oligomers and monomers). In addition, more and more data become available demonstrating that PHAs exhibit an intrinsic biological activity with respect to various cells and tissues in humans and laboratory animals.

All the main PHAs, both the synthetic (PLA, PGA, PLGA and PCL) and natural PHBs (PHBV, PHBHx, P4HB) ones, possess fairly good biocompatibility when compared to many other materials, which is sufficient for utilizing these biodegradable polymers to fabricate implants that come into contact with soft tissues, bones, and blood in compliance with ISO standards 10993 [2, 4, 17, 18]. However, a comparison of the tissue response to PHB and the synthetic polyesters PLA, PGA, or their copolymers, revealed that PHB elicits either a mild or moderate tissue response [2, 3], while PLA, PGA, and PLGA often induce chronic inflammation [18]. In most cases, PHB and its copolymers were characterized by good biocompatibility when used as implanted biomaterials [19–21]. The standard test for tissue reaction to subcutaneous implantation of PHB and its copolymers in film form, which is employed in the protocols of preclinical trials, reveals a mild or moderate response to the foreign material. A thin fibrous capsule (~ 100 μm) is formed during a month and is resorbed once the samples have biodegraded [19–22]. Many studies revealed a low lymphocyte count or virtually no lymphocytes (in particular, T lymphocytes) at the PHB insertion site, indicating that the immune reaction to this polymeric biomaterial is either significantly reduced or absent [23–26]. It was demonstrated that deeply purified PHB and PHBV also exhibit good hemocompatibility, so they can be used to produce blood-contacting medical devices: patches for the pericardial wall, the pulmonary artery, and the right atrium, as well as biodegradable coronary stents [25–30]. However, the biocompatibility of PHB is especially vividly witnessed when using PHB-based devices (e.g., porous scaffolds for bone tissue regeneration). Implantation of PHB-based devices into the area of bone tissue defect is not accompanied by the formation of a connective tissue capsule separating the polymeric material from the bone tissue, which is observed for many biomedical devices (e.g., those made of PLA). In other words, PHB becomes completely integrated into the bone tissue. Implantation of the PHB-based porous scaffold leads to vigorous vascularization of the scaffold and emergence of islets of the bone tissue newly formed from the granulation tissue in its pores [23, 24, 31]. Evaluation of the expression levels of various cytokines and other markers of inflammation in the implantation site of medical devices based on PHB (and its copolymer PHBV) revealed reduced expression levels of proinflammatory cytokines (interleukins, tumor necrosis factor, monocyte chemoattractant protein, inducible nitric oxide synthase, and C-reactive protein) compared to those for other materials and increased expression of genes encoding various proteins (type I collagen, caveolin-1, cytokeratin, heparan sulfate proteoglycan, thrombomodulin, and prostacycline), which are markers of regenerative processes taking place in cardiac, vascular, intestinal, neural, and osseous tissues [23, 25, 27, 28, 32–35]. However, chronic inflammatory response to the implantation of PHB-based devices (e.g., coronary stent prototypes) was observed in some cases. It should be mentioned that these devices were either fabricated through polymer melting or could have been insufficiently purified [36, 37]. Like during biodegradation, the method used for molding products made of a polymer, especially when applying extrusion or melt molding, may significantly affect the biocompatibility of PHB and its copolymers. Melting causes polymer recrystallization and abruptly slows down water diffusion in the polymer matrix, whereas water is a component that plays a crucial role in the formation of the PHB ultrastructure, which strongly affects its biological properties [38].

Due to its high biocompatibility, PHB is a promising material for use in cell biology and cellular engineering. Various mammalian cells (human and murine fibroblasts, rat, mouse, and human mesenchymal stem cells (MSCs), rabbit bone tissue osteoblasts, human osteogenic sarcoma cells, chondrocytes in rabbit articular cartilage and rabbit smooth muscle cells) exhibit good levels of cell adhesion, proliferation, and viability during *in vitro *cultivation on PHB-based films or porous scaffolds [3]. Nano- and microparticles of PHB and its copolymers have no cytotoxic effect on different cells at concentrations below 1 mg/ml [39, 40]; their endocytosis can be performed not only by macrophages, but also by osteoblasts, fibroblasts, and epithelial tumor cells [41–45]. Meanwhile, the cytotoxicity of PLA and PLGA nanoparticles was not detected only at concentrations below 66–100 μg/ml but was strongly marked at concentrations above 100 μg/ml [41, 46]. Water-soluble PHB oligomers consisting of ~ 25 3-hydroxybutyrate residues conjugated to lipoid acid also had no *in vitro *cytotoxic effect on keratinocytes at concentrations below 9 μg/ml [47].

**Fig. 3:**
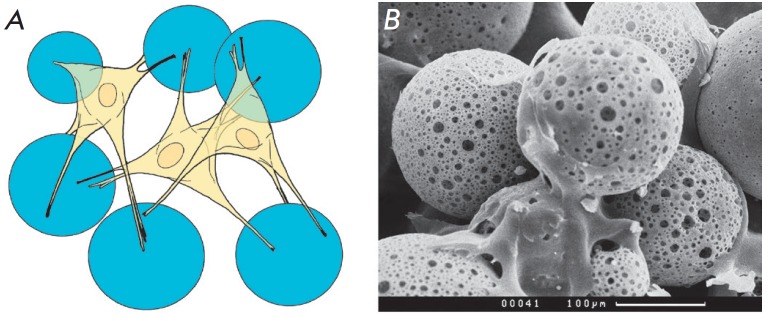
An *in vitro *experimental model of 3D cultivation of mesenchymal stem cells on PHB-based microspheres: scheme (A) and a SEM image (×300) (*B*) of cell growth on microspheres

Due to their high biocompatibility, PHB and other PHAs can be used to manufacture devices of various structures (porous matrices, microspheres, and scaffolds) for experimental modeling of the 3D growth of various human and mammalian cells (mesenchymal stem cells, fibroblasts, various tumor cell lines) under *in vitro *conditions, which will allow one to create experimental models of various diseases; cancer in particular [48] (*Fig. 3*). Meanwhile, one should bear in mind that characteristics of polymers such as their chemical composition, surface morphology, surface energy and hydrophobicity have a profound impact on cell viability and growth [49]: for example, chemical treatment of the surface of PHB-based items facilitates cell growth on them [3].

**Biodegradation of polyhydroxyalkanoates and their biological activity**

The biodegradation rate of widely used sPHAs, PLA, and PGA is significantly higher than that of other PHAs, since biodegradation takes place preferentially via hydrolytic destruction. This destruction mechanism of sPHAs is the reason behind the many problems associated with their medical application. Thus, the degradation products of PLA, PGA, and PLGA, which are formed during rapid hydrolysis, have no time to be taken up by the organism and pH decreases drastically near the implant. Chronic tissue irritation caused by reduced pH is considered a serious problem associated with the use of polymer implants based on PLA, PGA, and PLGA; an optimal solution to this problem still needs to be found [18]. Chronic inflammation in response to the destruction of polylactides and polyglycolides can be aggravated by the immune response to release non-stereoregular water-soluble oligomers, degradation products of polymers belonging to this class [18, 50]. The products of hydrolytic destruction of PLA and PGA were shown to be cytotoxic [18, 41, 46]. Dendritic cells that can be activated by PLGA significantly contribute to the triggering of an inflammatory reaction to this polymer after its implantation [51]. In particular, this inflammatory response is one of the reasons why biodegradation of intraosseous implants made from these polymers is slowed down as they are “preserved” in a connective tissue capsule, which causes various complications, such as implant migration to the bones, fistulization, implant failure, etc. [18]. Various sorts of precautions are used to eliminate chronic inflammation. Hence, anti-inflammatory drugs (dexamethasone or curcumin) [52, 53], antibodies specific to proinflammatory cytokines (interferon-γ) [54] are added to PLGA-based products, or mesenchymal stem cells are used [51].

Natural poly(3-hydroxyalkanoates) are much more resistant to hydrolysis in aqueous media [55], including in the presence of various esterases [55–57]. In living tissues, the biodestruction rate can be manifold higher than that in an aqueous medium under *in vitro *model conditions even in the presence of high concentrations of lipolytic enzymes (e.g., lipase) [22].

Recent data demonstrate that the biodegradation of PHB and its copolymers takes place predominantly through the phagocytic ability of specialized cells (macrophages), as well as foreign body giant cells (FBGCs) and osteoclasts. In other words, specialized biodegradation of these polymers takes place. The insertion of devices based on PHB and its copolymers into the organism results in the recruitment of macrophages in the damaged area, which densely cover the polymeric material as a connective tissue capsule is formed around it and are actively involved in polymer biodegradation. The polymeric biomaterial is exposed to the extracellular fluid and cells, which may lead to cleavage of micro- and nanoparticles, oligomers, and the monomers from it [3, 19, 57–59]. Cells cause superficial erosion of the polymer, without significantly altering its physicochemical properties, which takes place upon bulk hydrolytic destruction of the polymer. It was demonstrated that signs of erosion (erosion pits 20–50 μm in diameter) remained on the polymer surface after macrophages and FBGCs had been removed [25, 26, 60, 61]. The low biodestruction rate reduces the concentrations of degradation products near the implant; for PHB, the predominant degradation product is 3-hydroxybutyric acid, which is much weaker (p*K*α = 4.41) than lactic acid (pKα = 3.73), the main biodegradation product of PLA and PLGA. Therefore, biodegradation of PHB and its copolymers does not cause medium acidification [18–20].

Macrophages are simultaneously activated by the polymeric material, which contributes to their phagocytic activity [36, 40, 62]. Macrophage adhesion on the surface of the polymeric material plays an important role. Biodegradation of polymeric membranes was shown to take place only once macrophages have adhered to their surface. If macrophages are incapable of adhering to the membrane, polymer degradation does not occur [63]. Macrophages and osteoclasts tightly adhere to polymeric PHB films and proliferate on them [62]. The expression of two types of lipases significantly increased after 7- and 14-day contacts between PHB and animal tissues; enhanced expression of the same types of lipases was observed in the liver. Furthermore, increased synthesis of cleaving enzymes such as type 1 and type 2 lipases, amylase, chymotrypsin, and trypsin was observed in the gastric wall immediately at the site where tissues came into contact with a PHB-based patch [34]. Two enzymes cleaving PHB were found in rat tissues: liver serine esterase with maximum activity observed in an alkaline medium (pH 9.5) and kidney esterase active in a neutral medium [64]. Experiments involving low-molecular-weight PHB particles demonstrated that macrophages are involved in the biodegradation of PHB [40]. It was found that macrophages and fibroblasts (although to a lesser extent) can phagocytize PHB particles 1–10 μm in size. At high concentrations of PHB particles (> 10 μg/ml), phagocytosis is accompanied by a toxic effect and changes in the functional status of macrophages but not fibroblasts [40]. Meanwhile, the nanoparticles (15–250 nm in size) of PHB and its copolymers had no significant cytotoxic effect on macrophages even at such a high concentration as 1 mg/ml, unlike PLA nanoparticles [41]. Phagocytosis of PHB microparticles was accompanied by enhanced production of nitric oxide (NO) and tumor necrosis factor-α (TNFα) in the activated macrophages, while phagocytosis of a large amount of microparticles caused microphage death. It was also demonstrated that phagocytosis of PHB particles gradually decreases due to vigorous biodegradation of PHB [40]. It is an interesting fact that not only macrophages, but osteoblasts as well can be involved in *in vitro *endocytosis of microparticles consisting of low-molecular-weight PHB. Upon co-cultivation of these cells, the phagocytic ability of osteoblasts, as well as their osteogenic activity (alkaline phosphatase activity), was stimulated by macrophages phagocytizing polymeric microparticles [40].

Hence, the contact between living cells and polymers even characterized by high biocompatibility can be accompanied by a natural inflammatory response by the organism to the implantation of a foreign body and by the activation of macrophages and osteoclasts as they cleave the polymer. However, one should differentiate between this biological activity of PHAs and the intrinsic biological activity of polymers as related to their specific properties.

**Intrinsic biological activity of polyhydroxyalkanoates**

PHB and its copolymers seemingly also exhibit an intrinsic biological activity. As mentioned earlier, they activate immune cells upon implantation, thus inducing secretion of proinflammatory cytokines by these cells [34, 35]. This effect is typical of a regular tissue response to the implantation of almost any materials, especially biodegradable ones. It was demonstrated that PHB-based products (non-woven patches, porous scaffolds) facilitate the regeneration of tissues in different organs: osseous, cardiac and vascular, neural, and intestinal tissues. Application of PHB-based devices causes a high degree of vascularization in the area of tissue defect repair [23-28, 32, 35, 65]. It was shown using the critical (the parietal region of rat skull) and noncritical (rat femur) models of bone defects that PHB-based porous scaffolds facilitate bone tissue regeneration. Minimal tissue response to the implantation related to gradual bioresorption of polymeric material, vigorous vascularization of the matrix, and intergrowth of the newly formed bone tissue into the pores of the PHB-based scaffold were observed at all stages of bone defect regeneration. Expression of osteogenic markers (e.g., type I collagen) is also indicative of bone tissue regeneration in a PHB-based scaffold [23, 24]. We observed a uniform formation of nascent bone tissue over the entire volume of the porous biopolymeric scaffold in the form of islets rather than on the edges; meanwhile, a fibrous capsule did not form around the biopolymeric material, an indication of its complete integration with the bone tissue [24, 65]. All this demonstrates that PHB exhibits excellent biocompatibility with bone tissue and possess an osteoconductive and even osteoinductive potential. The biological activity of PHB and its copolymers produced via bacterial biosynthesis was attributed to the fact that the polymeric material could have been insufficiently purified to remove bacterial lipopolysaccharide or DNA. However, even the highly purified polymer can elicit a cellular response [36].

A biological activity of porous scaffolds based on PHB and its copolymers (PHBV, PHBHx, and PHBVHx) was also demonstrated at a cellular level *in vitro*. Thus, terpolymer PHBVHx stimulated the proliferation of *HaCaT *human kertinocytes grown on polymeric films produced via precipitation from a solution. Investigation of the mechanism of stimulation of cell proliferation using the nanoparticles of this biopolymer demonstrated that the addition of PHBVHx nanoparticles at a concentration of 0.02–0.1 g/l stimulates an elevation of the current of calcium ions into the cytoplasm, which is one of the key signaling pathways for the activation of cell division. The degradation product of PHA, monomeric 3-hydroxybutyrate (*D*-3-hydroxybutyric acid, 3HB), also independently induces the activation of *HaCaT *human keratinocytes and *L929 *murine fibroblasts when used at concentrations ranging from 0.01 to 0.1 g/l (0.1–1.0 mM), as it increases calcium ion concentration in the cytoplasm, and also suppresses fibroblast apoptosis and necrosis [66–68]. This activity of 3HB is not surprising, since this ketone body is a natural mammalian metabolite that displays a profound biological activity [16]. However, biological activity can be exhibited not only by 3HB, but also by PHA oligomers. Thus, PHB oligomers and their copolymers with 4-hydroxybutyrate and 3-hydroxyhexanoate (with a chain length of 20–25 monomer units) are not cytotoxic when used at concentrations < 20 μg/ml, stimulate proliferation and suppress apoptosis, calcium release into the cytoplasm, and the formation of cell–cell contacts between pancreatic beta cells in mice [69].

**Fig. 4:**
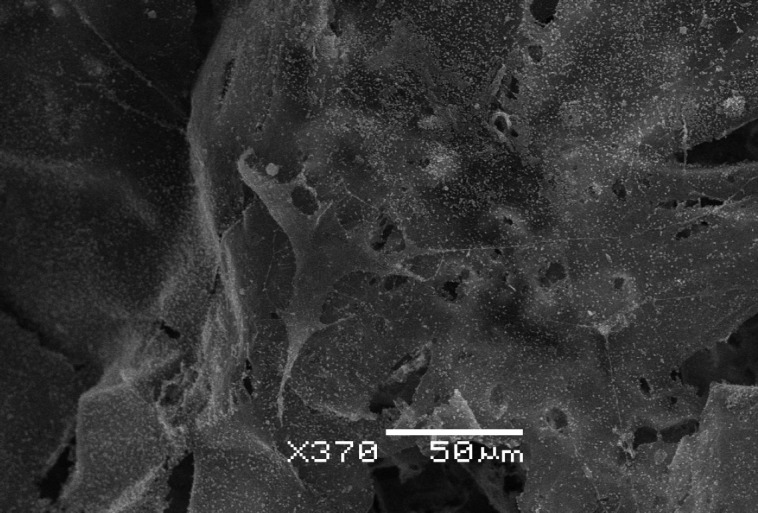
A mesenchymal stem cell with the osteoblast morphology on the PHB-based polymeric matrix (on the 21^st^ day of cultivation) and calcium salt deposits around it on the matrix. A SEM image (×370)

It was demonstrated that scaffolds based on PHB and its copolymers (PHBV, PHBHx) promote osteogenic differentiation of osteoblasts and mesenchymal stem cells in humans, rats, and rabbits (isolated both from the adipose tissue and from the bone marrow) when culturing the cells on these materials [23, 24, 39, 63, 70–72]. Differentiation of MSCs cultured on nPHAs-based scaffolds was confirmed by changes in cell morphology (*Fig. 4*), inhibition of their proliferation, increased alkaline phosphatase activity, calcium salt deposition in the cells [24, 39, 70–72], and expression of markers of osteogenic differentiation, and the formation of osseous tissue (alkaline phosphatase, type 1 collagen, *Runx2*, osteocalcin, and osteopontin) using the immunoenzyme techniques and PCR [23, 24, 70]. However, some studies failed to confirm the induction of osteogenic differentiation upon cultivation of embryonic progenitor populations on the scaffolds [73]. It is worth mentioning here that the growth and differentiation of MSCs can be affected by their physicochemical properties, as well as the microstructure and the topography of the devices made from the polymers used to culture the cells. This effect can even neutralize the influence of the bioactive molecules that stimulate cell growth or differentiation in a particular direction [49, 74, 75]. The impact of PHAs on MSC differentiation can also be related to the bioactivity of their biodegradation product, 3HB. Thus, 3HB at a concentration of 0.005–0.1 g/l (0.05–1 mM) causes osteogenic differentiation of MC3T3-E1 mouse osteoblasts, which was identified based on an elevation of the alkaline phosphatase activity, calcium deposition (Alizarin Red S staining quantification assay), and osteocalcin expression. The osteoinductive activity of 3HB was demonstrated *in vivo *for an osteoporosis model in female rats with their ovaries removed. Nevertheless, 3HB used at lower concentrations did not exhibit such an effect; slow biodegradation of PHAs gives rise to 3HB at concentrations much lower than 0.05 mM [76]. PHBHx also causes chondrogenic differentiation of MSCs, which was observed based on changes in the expression of chondrogenic genes acting as MSC markers (aggrecan, *col2*, *sox9*, *col10*, and *pthrp*) [74]. It was shown in other studies that PHB, PHBHx, PHBVHx, PHBO, and their composites, as well as PLA, stimulate neurogenic differentiation of MSCs, which can be observed based on changes in cell morphology and the expression of the genes coding for lineage-specific proteins (nestin, glial fibrillary acidic protein, and βIII-tubulin) [77, 78]. This theoretically could have been related to the neuroprotective effect of 3HB that was demonstrated earlier if it was not for the fact that the positive impact of 3HB on the nervous system is caused by the nutritional (energy) function of fatty acids, including 3HB, in neurons and manifests itself when these substances are used at extremely high doses [79]. However, it was also demonstrated that 3HB stimulates the formation of neuronal gap junctions for signal transduction, and this fact can be used to explain why this compound improves memory and learning ability [80]. It is interesting that the effects of PHAs on cell proliferation, differentiation, and apoptosis can be realized through integrins, the molecules that mediate cell–cell contacts and are involved in recognition. Differentiation of MSCs and osteoblast apoptosis proceed via the cascade mechanism initiated as PHAs interact with integrins on the cell surface [81, 82].

## EXPERIMENTAL

**Poly(3-hydroxybutyrate) as a reserve polymer in bacteria**

**Fig. 5:**
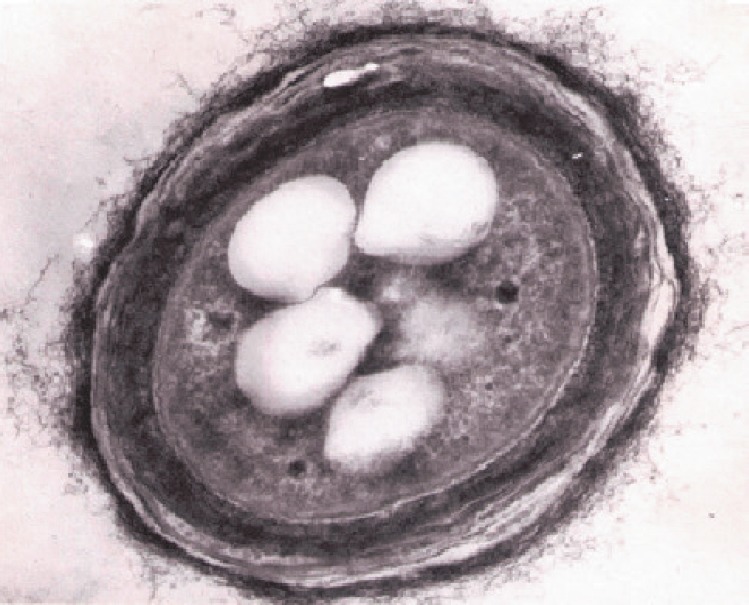
PHA-producing strain of *Azotobacter chroococcum *7B with PHB granules in a bacterial cell during polymer biosynthesis (TEM, ×50,000)

Natural poly(3-hydroxyalkanoates) have evolutionarily developed as reserve biopolymers, i.e., polymers that can be biodegraded by enzymatic systems in living organisms to release energy and carbon for cells to remain vitally active and ensure the biosynthesis of other biomolecules. The ability to synthesize reserve nPHAs, and PHB in particular, is commonly observed in prokaryotes; several hundreds of bacterial species utilize this biopolymer as a reserve compound. For most microorganisms, the accumulated nPHAs act as a source of carbon and energy if there is a lack thereof. Bacteria capable of synthesizing nPHAs accumulate the biopolymer in their cytoplasm as discrete inclusions (granules) that typically range between 100 and 800 nm in diameter (*Fig. 5*). The role of nPHAs, and PHB in particular, as a reserve material in bacteria was thoroughly discussed in the review by Anderson and Dawes [83].

Furthermore, human symbiotic and infectious bacteria, such as *Agrobacterium, Clostridium, Ralstonia, Bacillus, Burkholderia, Vibrio, Legionella, Pseudomonas, Mycobacterium, Acinetobacter, Sphin gomonas, Fusobacterium, Neisseria, Streptomyces, Bordetella, *and *Rickettsia *either are capable of synthesizing PHB or carry the enzymes (and genes encoding them) involved in its biosynthesis (primarily PHA polymerase). Some of these bacteria (e.g., *Pseudomonas sp.*) can synthesize both PHB and its various copolymers [84]. Many of these bacteria either constitute a significant portion of the normal human gut microbiota, which plays a crucial role in the formation of immunity and other organs (the oral cavity, the lungs, and the skin), or are causative agents of many common infectious diseases. Accordingly, the human immune system recognizes the antigens of these bacteria presumably from the very time the immunity starts to form during infancy. PHB is one of these common antigens familiar to the immunity. This probably is the reason for the high biocompatibility of this biopolymer and those of its synthetic analogs that have a similar structure and physicochemical properties. However, despite the fact that the immune system also comes into contact with lipopolysaccharide at the stage of immunity formation, this biopolymer is a potent immune stimulant. Meanwhile, PHB is also a product of symbiotic and infectious bacteria. It is quite possible that the function of PHB in the human body differs from that of the reserve material in microbiota.

**Endogenous poly(3-hydroxybutyrate) in animal tissues and its putative functions**

Contrary to the existing opinion that PHB is synthesized solely in prokaryotic cells, this biopolymer was discovered by Reusch [85] in almost all types of organisms. The short-chain complexed PHB (cPHB, ≤ 30 3-hydroxybutyrate monomers), and the medium-chain, or oligo-PHB (oPHB, 100–200 3-hydroxybutyrate monomers) were detected in various organs and tissues of mammals, including humans (as well as cow, sheep, and pig) and birds (chicken and turkey): in blood, brain, heart, liver, kidney, blood vessels, nerves, lipoprotein particles, platelets, etc. The cPHB/oPHB concentration varies from 3–4 μg/g in neural tissues and the brain; to 12 μg/g in blood plasma. The oPHB concentration in human blood plasma can vary in a rather wide range: between 0.6 and 18.2 μg/ml, the average value being 3.5 μg/ml [85]. It should be mentioned that 3HB, the intermediate product of PHB biodegradation, is a so-called ketone body. It is found in mammalian blood and tissues at a normal level of 0.3–1.3 mM and at much higher levels in pathology [86].

Reusch [85] suggested that besides acting as a reserve material and an energy depot in bacteria, PHB also plays different regulatory functions in eukaryotes and prokaryotes. PHB (namely, the short-chain cPHB and oPHB) affects the function of protein receptors and channels, as well as DNA, by forming noncovalent or covalent bonds with them. The researchers attributed the presence of PHB in different human tissues to the existence of some biochemical synthesis mechanisms of this biopolymer. They showed that cPHB and oPHB form noncovalent complexes with inorganic polyphosphates and calcium ions, which can function as nonprotein channels that allow inorganic ions to pass through the cell membrane. These structures also form noncovalent complexes with ion-channel proteins and are their components. They also affect the receptor and channel functions through covalent binding. Thus, PHB oligomers bind covalently to calcium ATPase in the cell membrane of human red blood cells and simultaneously form a complex with inorganic phosphates [86]. Indirect evidence has been obtained showing that PHB–protein conjugates play some physiological role. Thus, conjugation of DP18L antitumor peptides to 3-hydroxydecanoate enhances their activity [87].

**Putative functions of poly(3-hydroxybutyrate) in the microbiota of animals**

However, PHB can have other functions in the human body that do not require its synthesis. It is fair to assume that PHB is somehow involved in the interplay between gut bacteria, where this biopolymer is synthesized, immune cells, and the intestinal epithelium. This hypothesis is supported by the special role played by PHB in the symbiosis between the gut bacteria and the host organism. For example, the synthesis of PHB contributes to the interaction between *Burkholderia *bacteria and their host, the bean bug *Riptortus pedestris*, making these bacteria more resistant to the immune system of this bug [88]. It was also demonstrated that the biosynthesis of PHB plays a crucial role in the microbiota of sea cucumber *Apostichopus japonicus*. The synthesis of PHB seems to modulate the intestinal microbiota of the sea cucumber, which increases the animal’s size manifold [89]. The study focused on the ability of histamine to regulate the synthesis of low-molecular-weight cPHB in *Escherichia coli *deserves close attention. Histamine plays an important role as a means of communication between bacteria and the host organism; it also regulates the intestinal immunity, so that the bacteria are recognized as “self” by the host organism. Therefore, the effect of histamine on the synthesis of cPHB may indicate that this biopolymer is involved in adaptation and coexistence with the host organism [90]. Furthermore, it was demonstrated that PHB is effective in treating infectious diseases: giving PHB as food to brine shrimp *Artemia nauplii *protected them against a disease caused by *Vibrio campbellii*; the effectiveness of PHB was 100-fold higher than that of 3-hydroxybutyric acid [91]. Furthermore, PHB can inhibit not only *Vibrio *sp*.*, but also *E. coli *and *Salmonella *sp*. *[92]. It was also demonstrated that the biodegradation products of some nPHAs (e.g., 3-hydroxyoctanoate) exhibit an antimicrobial activity with respect to a number of Gram-negative and Gram-positive bacteria, as well as inhibit the production of the metabolites associated with the pathogenic activity of these bacteria, while a much higher nPHA concentration is needed for a cytotoxic effect on human fibroblasts [93].

An interesting fact indicating that 3-hydroxybutyrate dimers and trimers are sex pheromones in spiders also indicates that PHB might possess some signaling functions in the organism [94]. It is quite possible that these pheromones can be products of the biosynthesis performed by bacteria in the microbiota of arthropod species. Thus, in *Costelytra zealandica *beetles, sex pheromone is phenol synthesized from tyrosine by symbiotic bacteria *Morganella morganii *in special glands [95]. 3-Hydroxybutyrate dimers and trimers were found in fungus *Hypoxylon truncatum*; however, the mechanism underlying their synthesis is yet to be established [96].

P4HB and PHB4HB are used to manufacture a number of biodegradable medical devices: surgical suture material, woven mesh endoprostheses and plug endoprostheses, as well as scaffolds for soft tissue regeneration. Due to its modified chemical structure, P4HB (as well as PLA and PGA) preferentially undergoes hydrolytic degradation. These polymers do not exist in nature; they are obtained through bioengineering via biosynthesis by the genetically modified *E. coli *strain K12. P4HB monomer, 4-hydroxybutyrate (γ-hydroxybutyric acid), similar to 3-hydroxybutyrate, is a natural metabolite and one of the neurotransmitters used as a potent psychoactive agent and even listed in the controlled drugs register [97].

## CONCLUSIONS

**Table:** The biological activity of synthetic and natural PHAs in the human body and the natural functions of poly(3-hydroxybutyrate)

Biological activity	Potential causes	Natural functions
Activation of macrophages and osteoclasts [36, 40, 63].	The ability to undergo hydrolytic and enzymatic destruction [1–4, 19, 58–60]. Preferentially cellular biodegradation of nPHAs [25, 26, 61, 62].	The ability of PHB to undergo controlled biodegradation (as an intracellular reserve material in bacteria) [83].
Stimulation of proliferation of cells (keratinocytes, fibroblasts, and beta-cells) [67–70].	Intrinsic biological activity of nPHAs [69, 81, 82] and 3HB [67, 68].	The potential signaling function of PHB upon the interplay between gut bacteria synthesizing it and the immune cells and intestinal epithelial cells [88–90, 94]. The potential functionality of endogenous PHB [85]. Various functions of the ketone body 3HB and other 3-hydroxyalkanoates in the mammalian organism [76, 79, 93].
Stimulation of osteogenic, chondrogenic, and neurogenic differentiation of osteoblasts and MSCs [23, 24, 39, 63, 70–72, 77, 78].	Physicochemical properties of PHAs [24, 49, 71], the microstructure and topography of medical products [74, 75], biodegradability [1–4], intrinsic biological activity of nPHAs [69, 81, 82] and 3HB [77, 80].	
Activation of regeneration of various tissues (cardiac and vascular, intestinal, neural, and osseous) [1, 2, 4, 23, 25, 27, 28, 32–35, 36]		
Chronic inflammatory response (low [1–4, 19–35], pronounced [18, 36, 37]).	Acidification of tissues with biodegradation products of PLA [18], immune response to sPHAs with the modified chemical structure [18, 50, 51], insufficient purification, harsh treatment of polymers (e.g., by melting) [38, 49], the microstructure and shape of medical devices [18].	Low toxicity of PHB as an intracellular reserve material in bacteria [83]; low immunogenicity of PHB due to its presence in mammalian gut microbiota [84] and potential presence of endogenous PHB in mammals [85].
Cytotoxicity (low [1–4, 39–45, 47], pronounced [18, 41, 46]).	Acidification of tissues with biodegradation products of sPHAs [18, 41, 46].	

Hence, the biological activity of PHAs observed by many researchers (e.g., the ability of these polymers to stimulate the regeneration of bone and cartilage tissues) is related not only to the physicochemical properties of PHAs or to the structure of items based on these polymers, but also to the fact that PHAs exhibit an intrinsic activity, which is in turn caused by the natural functions of PHB, a precursor used to produce these polymers (*Table*). This relationship is also observed between the biodegradability of PHAs used to fabricate medical devices in human tissues and the natural function of PHB as a reserve biopolymer in bacterial cells, since the reserve material must be able to undergo cleavage by cellular enzymes in order to be able to perform its function. This biopolymer possibly has certain signaling functions in our organism through which the gut bacteria interact with the immune cells, intestinal mucosa, and other tissues by eliciting a certain physiological response in them. It is fair to assume that the structure of PHAs produced by both chemical synthesis and bioengineering is similar to that of PHB, thus making it possible to mimic the biological properties of PHB related to the functions acquired by this biopolymer during the long-term evolution of the organisms in which it is synthesized.

Although the overwhelming majority of devices and pharmaceuticals based on PHAs were produced from synthetic PHAs, several products based on natural PHAs have already been designed and are used in practice: e.g., the ElastoPHB biopolymer membrane system for repairing soft and cartilage tissue defects (BIOMIR Service JSC, Krasnoznamensk, Russia) [98] and Phasix™ Plug TephaFLEX composite mesh endoprosthesis (Tepha Inc., USA) [97] (*Fig. 2Fig. 2*). The bioengineering plant belonging to the Italian company Bio-on (http://www.bio-on.it/index.php) that is currently under construction and is intended for large-scale industrial production of PHB and its copolymers also justifies the never-abating interest in natural PHAs used both in industry (packaging, textile, cosmetics, and household goods) and in medicine.

Hence, the field of science discussed in this review requires further comprehensive and meticulous research, which will allow us to uncover the natural functions of the polymers used in medicine (the biomimetic analogs of natural predecessors) and to design novel, nature-like technologies for producing polymer-based medical items and next-generation drugs.

## Abbreviations

PHAs: polyhydroxyalkanoates
sPHAs: chemically synthetized polyhydroxyalkanoates
nPHAs: natural polyhydroxyalkanoates, poly(3-hydroxyalkanoates)
PLA: poly(2-hydroxypropanoic) (polylactic) acid, polylactide
PGA: poly(2-hydroxyacetic) (polyglycolic) acid, polyglycolide
PLGA: poly(lactic-co-glycolic) acid (polylactide-co-glycolide)
PCL: poly(6-hydroxycaprolactone) (poly(ε-hydroxycaprolactone))
PDS: poly(p-dioxanone)
PHB: poly(3-hydroxybutyric) acid (poly(3-hydroxybutyrate))
cPHB: short-chain complexed endogenic PHB
oPHB: medium-chain or oligo-PHB
P4HB: poly(4-hydroxybutyric) acid (poly(4-hydroxybutyrate))
PHV: poly(3-hydroxyvaleric) acid (poly(3-hydroxyvalerate))
PHBV: poly(3-hydroxybutyrate-co-3-hydroxyvalerate)
PHHx: poly(3-hydroxyhexanoate)
PHBHx: poly(3-hydroxybutyrate-co-3-hydroxyhexanoate)
PHBVHx: poly(3-hydroxybutyrate-co-3-hydroxyvalerate-co-3-hydroxyhexanoate)
PHBO: poly(3-hydroxybutyrate-co-3-hydroxyoctanoate)
PHB4HB: poly(3-hydroxybutyrate-co-4-hydroxybutyrate)
3HB: 3-hydroxybutyrate; FBGCs – foreign body giant cells
NO: nitric oxide
TNF-α: tumor necrosis factor alfa
MSCs: mesenchymal stem cells
